# Acute pancreatic panniculitis in a domestic ferret (*Mustela putorius furo*): diagnostic insights and a potential drug-induced etiology

**DOI:** 10.1007/s11259-025-10970-y

**Published:** 2025-11-11

**Authors:** Jacobo Giner, José Villora, Carles Juan-Sallés, Ana Rodriguez-Largo, Álex Gómez, Sergio Villanueva-Saz, Diana Marteles

**Affiliations:** 1 Menescalia Veterinary Clinic, Ismael Merlo, 5, 46020 Valencia, Spain; 2 Departamento de Medicina y Cirugía Animal, Facultad de Veterinaria, Universidad Cardenal Herrera-CEU, CEU Universities, Valencia, 46115 Spain; 3 Clinical Immunology Laboratory, Veterinary Faculty, University of Zaragoza, Calle Miguel Servet, 177, Zaragoza, 50013 Spain; 4 Department of Animal Pathology, Veterinary Faculty, University of Zaragoza, Calle Miguel Servet, 177, Zaragoza, 50013 Spain; 5 Noah’s Path, Arquitecto Santiago Pérez Aracil 30 bajo (centro veterinario), Elche, 03203 Spain; 6 Servei de Diagnòstic Patologia Veterinària, Departament de Sanitat I Anatomia Animal, Universitat Autònoma de Barcelona (UAB), Cerdanyola del Vallès, 08193 Spain; 7 Instituto Agroalimentario de Aragón-IA2 (Universidad de Zaragoza-CITA), Calle Miguel Servet, 177, Zaragoza, 50013 Spain

**Keywords:** Acute pancreatic necrosis, Antibiotic, Ferret, Panniculitis, Spain

## Abstract

Panniculitis is a rare condition in ferrets (*Mustela putorius furo*), previously linked to nutritional deficiencies, infections, trauma, and injections. Pancreatic panniculitis, caused by the systemic release of pancreatic enzymes during pancreatic injury, has been documented in humans and other animal species, but not in ferrets. This report describes the first known case in a domestic ferret. A 2-year-old male ferret presented with ulcerated cutaneous nodules predominantly affecting the hind limbs and inguinal abdominal region. The ferret had been treated with rifampicin and clarithromycin for suspected mycobacterial infection. Hyperglycemia, hyperglobulinemia, and elevated alkaline phosphatase were noted. Cytology and culture of the lesions revealed neutrophilic inflammation and *Pseudomonas aeruginosa*, respectively. The animal developed acute abdominal discomfort and died shortly after. Post-mortem examination revealed severe pancreatic necrosis and peripancreatic panniculitis, with splenic pyogranulomatous inflammation. Immunohistochemistry detected systemic coronavirus antigen (clone FCV3-70) only in the spleen. In this patient, prolonged use of rifampicin and clarithromycin is considered a potential contributing factor to the development of acute pancreatic necrosis. This case highlights the clinical relevance of pancreatic panniculitis in ferrets and suggests a possible association between extended rifampicin/clarithromycin therapy and pancreatic necrosis. Ultrasonography and serum glucose, lipase, and amylase measurements are recommended for early diagnosis. Histopathological evaluation remains essential for confirmation.

## Background

Panniculitis is defined as inflammation of the panniculus adiposus, the adipose tissue located within the subcutaneous layer. Although panniculitis is a rare diagnosis in ferrets, several etiologies have been reported, including vitamin E deficiency (Brooks et al. 1985), *Pseudomonas luteola* infection (Baum et al. 2015), *Leishmania infantum* (Giner et al. 2024), *Cryptococcus* spp. (Halck et al. 2023), and *Mycobacterium* spp. (Lucas et al. 2000). Moreover, panniculitis may occur secondary to trauma, burns, post-surgical complications, the presence of sharp foreign bodies, or injections (O´Kell et al. 2010).

Pancreatic panniculitis has been described in humans (Zundler et al. 2016), dogs (Mellanby et al. 2003; Muller et al. 2022), cats (Fabbrini et al. 2005), and horses (Waitt et al. 2006). In this condition, the release of pancreatic enzymes from damaged exocrine pancreatic tissue induces necrosis of the peri-pancreatic adipose tissue. These enzymes can enter the bloodstream and reach the subcutaneous fat, where they elicit a severe inflammatory response (Marcos et al. 2017; Zundler et al. 2016; Seguí et al. 2023). Polyarthritis and osteomyelitis have also been reported in association with exocrine pancreatic injury in both humans and dogs (Gear et al. 2006; Zundler et al. 2016). Pancreatic panniculitis may result from acute pancreatitis (Mellanby et al. 2003; Waitt et al. 2006; Muller et al. 2022) or pancreatic neoplasia (Fabbrini et al. 2005; Gear et al. 2006).

Due to the absence of published information on this condition in ferrets, there is a significant gap in the current understanding of its presentation and diagnosis in this species. To the best of the authors’ knowledge, this is the first report describing pancreatic panniculitis in a ferret. Additionally, this case provides valuable insights into the diagnosis of acute pancreatitis in this species.

## Case presentation

A 2-year-old intact male domestic ferret from Valencia, Spain, was clinically evaluated due to a 5-day history of multiple ulcerated cutaneous nodules located on the hind limbs, perineal region, and groin. The ferret had been adopted at six months of age and lived exclusively indoors. Prior to the onset of panniculitis, the ferret had been receiving antibiotic therapy with rifampicin (15 mg/kg, SID) and clarithromycin (15 mg/kg, TID) for three months, based on a high index of suspicion for mycobacterial infection. This empirical treatment was initiated after other systemic diseases were ruled out through a comprehensive clinical workup. This diagnostic process included a complete medical history, physical examination, a serial complete blood cell counts and biochemical profiles including protein electrophoresis which revealed chronic severe hyperglobulinemia with hypergammaglobulinemia, thoracic radiography, abdominal ultrasonography, and cytological evaluation of a mediastinal pyogranulomatous lymph node via ultrasound-guided fine-needle aspiration. Additionally, molecular testing yielded negative results for Aleutian disease virus and ferret systemic coronavirus (FRSCV) by qPCR, and *L. infantum* serology was negative using an in-house western blot technique (Giner et al. 2024). These results supported the presumptive diagnosis of a mycobacterial infection, as alternative causes of severe hyperglobulinemia and pyogranulomatous lymphadenitis commonly reported in domestic ferrets had been excluded prior to initiation of antimycobacterial therapy. Specific diagnostic tests for mycobacteriosis, including PCR and culture, were not performed because the owners declined other sample collection of the mediastinal lymph nodes due to the potential risks associated with the procedure.

**Fig. 1:**
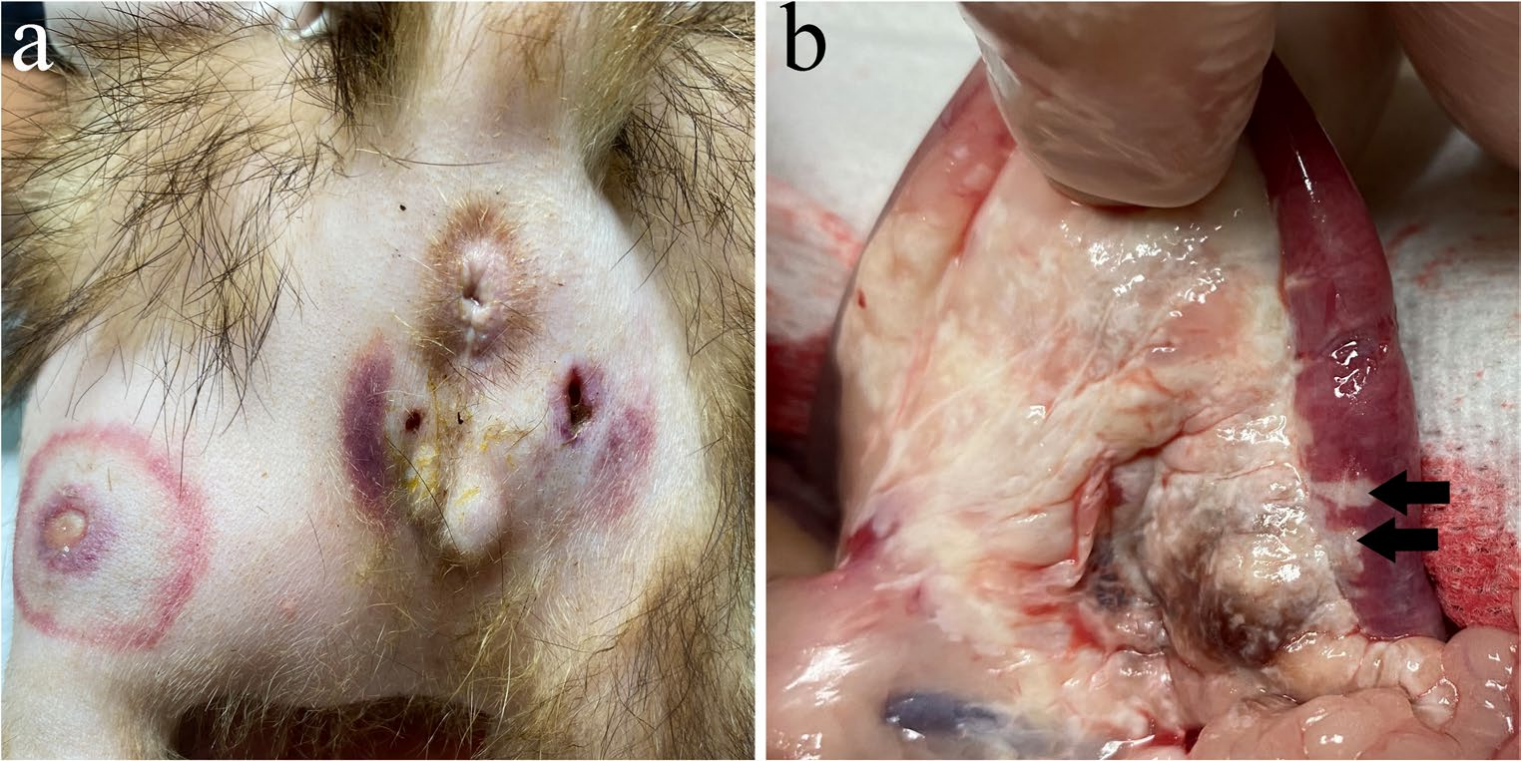
Ferret with pancreatic panniculitis. (**a**) Multifocal necrotizing-ulcerative dermatitis. (**b**) Diffuse necrosis involves the pancreas and peripancreatic adipose tissue, with multifocal extension into the duodenal wall

On physical examination when the patient was presented with dermatological lesions, the ferret was in good body condition, active, alert, normothermic, and well-hydrated. Multiple well-circumscribed areas of cutaneous erythema with central ulceration and purulent discharge were observed affecting the hind limbs and inguinal abdominal region. (Fig. 1a). These lesions were typically surrounded by a concentric ring of indurated tissue. Skin scrapings were collected from the lesions and examined cytologically using Diff-Quik staining. In addition, aerobic and anaerobic bacterial culture and antibiotic sensitivity testing were performed on the purulent discharge. A complete blood count and serum biochemistry panel were also conducted, including measurements of alanine aminotransferase (ALT), alkaline phosphatase (ALP), bilirubin, creatinine, blood urea nitrogen (BUN), total protein, albumin, globulins, glucose, cholesterol, calcium, and phosphorus. Pending culture results, empirical antibiotic treatment with amoxicillin/clavulanate (15 mg/kg, BID PO) was initiated and previous antimicrobial therapy was discontinued.

On the following day, deep excisional biopsies of skin lesions were obtained and the patient was discharged from hospital care tolerating oral intake. A few hours later, the patient became recumbent, semi-comatose, and exhibited signs consistent with abdominal pain, including bruxism, back arching and vocalization upon abdominal palpation. Abdominal palpation revealed a firm mass in the right cranial abdominal quadrant. A continuous intravenous infusion of fentanyl (5 µg/kg/h) and lidocaine (1 mg/kg/h) was initiated for analgesia, and an abdominal ultrasound was performed. The ultrasonographic examination revealed multifocal hypoechoic areas, hyperechoic regions, and mixed echogenic patterns throughout the pancreatic region, findings consistent with severe pancreatic pathology. An emergency exploratory laparotomy was recommended; however, the patient died during the pre-anaesthetic preparation.

A gross post-mortem examination was performed by the referring clinician. Tissue samples from the pancreas, duodenum, spleen, and skin lesions were fixed in 10% neutral-buffered formalin and submitted for histopathological evaluation. No other tissue samples were obtained due to normal macroscopical appearance. Representative sections were embedded in paraffin, sectioned at 5 μm, and stained with haematoxylin and eosin (H&E).

Immunohistochemistry (IHC) for coronavirus antigen was performed on the previously collected tissue samples using the PT-Link Automated System (Dako, www.agilent.com) for deparaffinization, rehydration, and epitope retrieval. Immunolabelling was carried out on an Autostainer Plus (Dako) following the manufacturer’s protocols, including the use of proprietary buffers and solutions.The primary antibody, a mouse monoclonal antibody against feline coronavirus (clone FCV3-70; Custom Monoclonals International, www.vetmabs.com), previously shown to cross-react with ferret systemic coronavirus (FRSCV), was applied at a dilution of 1:500. The Rabbit/Mouse EnVision Detection System (K5007; Dako) was used according to the manufacturer’s recommended dilution. After washing, slides were incubated for 5 min with the DAB + Substrate Buffer and Dako Liquid DAB + Chromogen System (K3468; Dako) to visualize antigen binding. Slides were then counterstained with Mayer’s haematoxylin for 10 s, rinsed in running tap water, and subsequently dehydrated, cleared, and mounted automatically.A section of tissue from a cat diagnosed with feline infectious peritonitis served as a positive control. Negative controls consisted of tissue sections processed without the primary antibody.

The complete blood cell count revealed a decreased red blood cell count (5.31 M/µL; reference range [RR]: 6.6–12.18 M/µL), haematocrit (24.3%; RR: 36.6–54.5%), and hemoglobin concentration (9.7 g/dL; RR: 11.2–17.3 g/dL). Serum biochemistry showed hyperglycemia (263 mg/dL; RR: 94–207 mg/dL), a markedly elevated alkaline phosphatase level (445 U/L; RR: 9–84 U/L), and mild hypocalcemia (7.6 mg/dL; RR: 8–11.8 mg/dL). Serum protein electrophoresis revealed hyperproteinemia (8.0 g/dL; RR: 5.2–7.3 g/dL), characterized by marked hyperglobulinemia (5.6 g/dL; RR: 1.8–3.1 g/dL).

Cytological evaluation of the cutaneous lesions revealed numerous degenerated neutrophils admixed with colonies of extracellular bacilli. Bacterial culture of the lesions identified *Pseudomonas aeruginosa*.

**Fig. 2:**
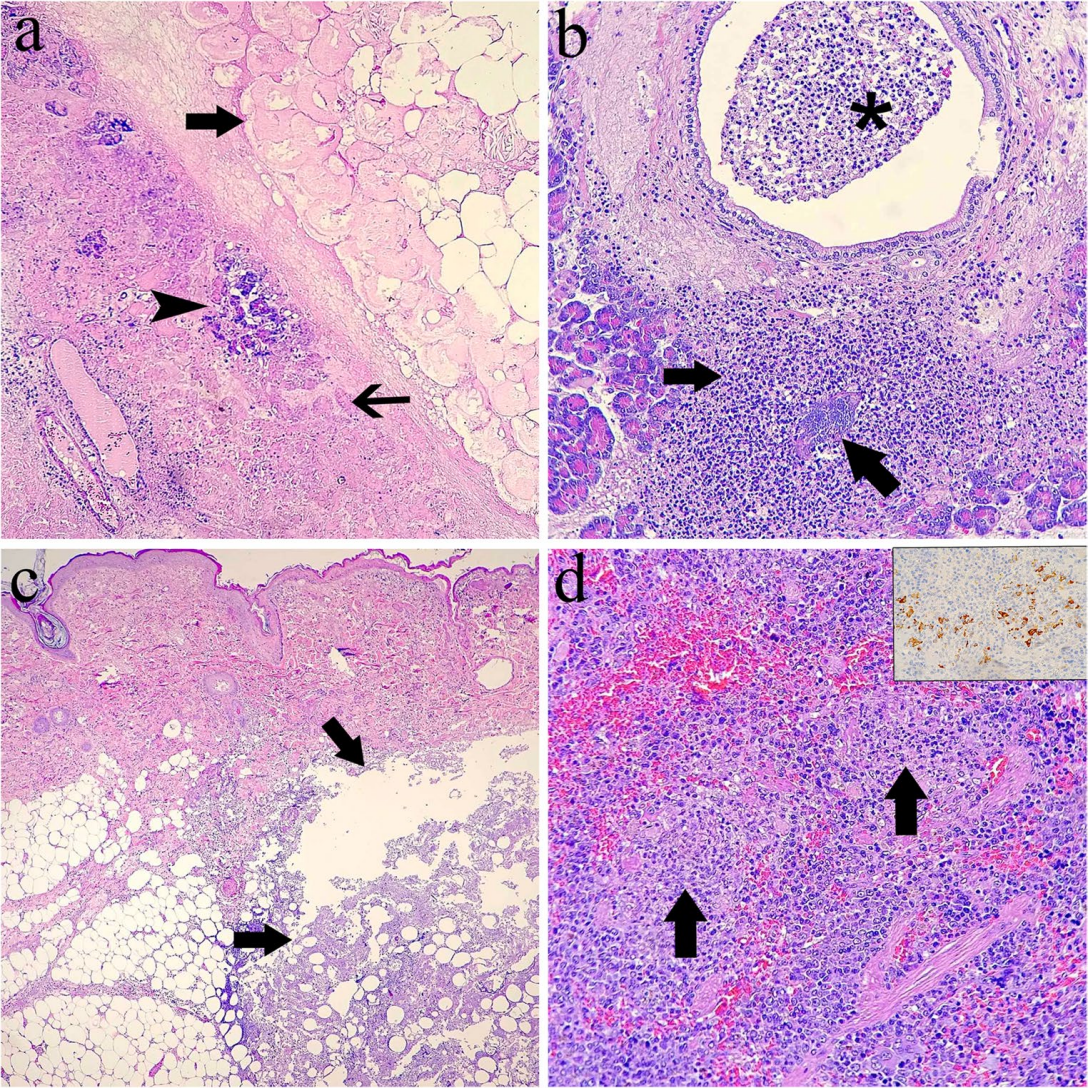
Ferret with pancreatic panniculitis. (**a**) Pancreatic tissue is almost completely necrotic (thin arrow). Few pancreatic acini remain unaffected (arrowhead). Peripancreatic adipose tissue is also necrotic (arrow). Hematoxylin-eosin (HE). (**b**) A focus of suppurative inflammation with an intralesional bacterial colony (arrow) in the pancreatic acinar tissue is adjacent to a duct plugged with a luminal exudate of neutrophils (asterisk). (**c**) The panniculus contains a large focus of necrosis and suppurative inflammation (arrows). HE. (**d**) Two foci of pyogranulomatous inflammation (arrows) are present in the red pulp, which is diffusely colonized by abundant hematopoietic precursors. HE. Inset: splenic macrophages showing an immunoreactivity labelling. Immunohistochemistry, coronavirus antigen

On gross post-mortem examination, the peripancreatic adipose tissue and pancreas were diffusely whitish and increased in consistency, with whitish areas extending multifocally into the wall of the duodenum (Fig. 1b). The spleen was enlarged and had an irregular surface and increased consistency. Microscopically, severe necrosis of the pancreas and peri-pancreatic adipose tissue was noted, with mild infiltration of neutrophils, accumulation of cellular debris and prominent proteinaceous oedema expanding the interlobular septum (Fig. 2a). Necrosis involved blood vessels and ducts, and extended into the duodenum up to the mucosa. Occasional colonies of cocci were observed in necrotic ducts or vessels. A section of the pancreas was not necrotic and exhibited multifocal infiltration by large numbers of neutrophils. The inflammatory infiltrate occasionally extended into the duct walls and lumina, and within one of these foci, a colony of coccoid bacteria was observed (Fig. 2b). The cutaneous lesions noted clinically consisted of large areas of necrosis and neutrophilic inflammation in the adipose tissue (Fig. 2c) with involvement of the vascular wall and extension into the subjacent skeletal muscle. The spleen contained numerous small infiltrates of neutrophils occasionally surrounded by macrophages (Fig. 2d) and deposits of fibrin scattered in the red pulp. The wall of some splenic veins was infiltrated with numerous neutrophils, lymphocytes and plasma cells.

IHC for coronaviral antigen revealed cytoplasmic immunolabelling of scattered macrophages within foci of pyogranulomatous splenitis (Fig. 2d, inset). No coronaviral antigen was present in the pancreas, peripancreatic adipose tissue and duodenum. Likewise, no pyogranulomatous lesions suggestive of mycobacterial infection were identified in the tissues analyzed.

## Discussion and conclusions

This is the first reported case of pancreatic panniculitis in a domestic ferret. Pancreatic panniculitis has been well documented in humans (García-Romero and Vanaclocha 2008; Zundler et al. 2016), dogs (Mellanby et al. 2003; Muller et al. 2022), cats (Fabbrini et al. 2005), and horses (Waitt et al. 2006). This condition has been associated with both acute pancreatitis (Mellanby et al. 2003; Waitt et al. 2006; Muller et al. 2022) and pancreatic neoplasia (Fabbrini et al. 2005; Gear et al. 2006). In the present case, the panniculitis was secondary to acute pancreatic necrosis of unknown etiology.

Pancreatitis not associated with partial pancreatectomy or FRSCV infection is considered an uncommon finding in ferrets (Sulkosky et al. 2025). In this case, the patient exhibited foci of suppurative pancreatitis with intralesional bacteria within non-necrotic pancreatic tissue, which was interpreted as a probable secondary infection ascending from the duodenum. Although pyogranulomatous pancreatitis has been associated with FRSCV infection in ferrets (Garner et al. 2008; Wills et al. 2018), immunohistochemistry for coronavirus antigen was negative in both the pancreas and peripancreatic adipose tissue. Consequently, systemic coronavirus infection was interpreted as a concurrent but unrelated disease process. In dogs and cats, pancreatic necrosis has been linked to various causes including trauma, exposure to toxins, corticosteroid use, pancreatic duct obstruction, high-fat diets, and obesity (Forman et al. 2021; Cridge et al. 2022). Based on a comprehensive review of the clinical history, these potential causes were excluded, supporting drug-induced acute pancreatitis as the most plausible etiology of the pancreatic lesions observed in this patient.

Currently, there is no single biochemical test that can be considered the “gold standard” for the diagnosis or assessment of the severity of acute pancreatitis. In human medicine, serum amylase and lipase remain important biochemical markers for the diagnosis of acute pancreatitis (Yadav et al. 2002). However, in the present case, serum amylase and lipase were not evaluated. Imaging techniques such as ultrasonography and computed tomography are also essential diagnostic tools for assessing pancreatic pathology (Szatmary et al. 2022). Recently, experimentally induced acute pancreatitis in ferrets has been associated with dysfunction of the islets of Langerhans and significant hyperglycaemia (Yi et al. 2021). In the current case, the ferret exhibited marked hyperglycaemia, suggesting that serum glucose measurement may have diagnostic value in cases of acute pancreatic necrosis in this species. Nevertheless, definitive diagnosis of acute pancreatitis relies on histopathological evaluation of pancreatic tissue.

In conclusion, this report describes, for the first time, pancreatic panniculitis in a ferret (*Mustela putorius furo*). For early diagnosis of acute pancreatitis in this species, abdominal ultrasonography along with measurement of serum glucose, lipase, and amylase is recommended. In this case, prolonged treatment with rifampicin and clarithromycin is suggested as a possible contributing factor to the development of acute pancreatic necrosis in ferrets.

## Data Availability

The data that support the findings of this study are available from the corresponding author upon reasonable request.
